# Recent insights into crosstalk between genetic parasites and their host genome

**DOI:** 10.1093/bfgp/elac032

**Published:** 2022-10-28

**Authors:** Amit K Mandal

**Keywords:** transposable elements, host genome, functional genomics, genome integrity, immune context

## Abstract

The bulk of higher order organismal genomes is comprised of transposable element (TE) copies, i.e. genetic parasites. The host–parasite relation is multi-faceted, varying across genomic region (genic versus intergenic), life-cycle stages, tissue-type and of course in health versus pathological state. The reach of functional genomics though, in investigating genotype-to-phenotype relations, has been limited when TEs are involved. The aim of this review is to highlight recent progress made in understanding how TE origin biochemical activity interacts with the central dogma stages of the host genome. Such interaction can also bring about modulation of the immune context and this could have important repercussions in disease state where immunity has a role to play. Thus, the review is to instigate ideas and action points around identifying evolutionary adaptations that the host genome and the genetic parasite have evolved and why they could be relevant.

## Introduction

Transposable elements (TEs) have been termed ‘selfish genes’ because while the host genome genes together work towards cellular homeostasis, TEs can have their own separate agenda [1]. Whereas the host genome genes have defined locations, TEs were first discovered for their transposition ability [2]. Besides, they can carry their own promoter and are transcriptionally active to varying degree and some TEs even code for protein products, essentially enzymes needed to achieve mobility around the genome [3]. Under the umbrella term of TE, there are different families that have distinctive features in terms of phylogenetic origin, life-cycle mechanism and more intriguingly tissue specificity and/or an activity profile linked to cellular cues such as developmental state, stress or immune context. The reader is referred to these excellent reviews on genome organisation, TE classification and distribution trend across the genome [4–6]. The biochemical activity arising from interaction between TEs, and various regulatory modules of the host genome could sometimes be beneficial for the host (cell or gene, or both), sometimes detrimental and in all probability, due to the sheer extent of such interaction events, sometimes neutral [7]. Thus, the term ‘genetic parasites’ (as opposed to ‘selfish genes’) for TEs appears better in being able to accommodate the different outcomes of host genome-to-TE interaction.

At present, our understanding of the central dogma does not offer us a predictability power to assign beforehand the outcome label to different host genome-to-TE interactions. More than half-a-century of functional genomics into protein-coding genes has allowed us to create annotation framework such as gene ontology and in the past decade or so, a preliminary understanding of tentative functions for non-coding RNAs too [8, 9]. But we are nowhere near to structure and create a hierarchical framework such as GO for TEs too. And the reason is that whereas there are ~20 000 protein-coding genes in the human genome, TEs are present in millions of copies [10]. Furthermore, protein-coding and many non-coding RNAs too are constituted, sequence-composition-wise, uniquely. Protein domains could be shared between different protein-coding genes, sequence moieties within untranslated regions (UTRs) (of mRNAs) could also be shared, but overall a given gene body (exons + introns) is a unique entity within the genome. One exception to uniqueness in terms of sequence composition could be pseudogenes, but such derived gene bodies for a given parent gene are limited in numbers [11]. On the other hand, TEs within a given family are sequence-composition wise redundant/repetitive, could amount to millions of copies and hence widely called ‘repeat elements’ (though this term also comprises microsatellites and simple-sequence repeats that are not transcriptionally active) [12, 13].

In this review, the aim is to highlight the overlapping nature of the genome regulatory networks that enable co-existence of TE copies alongside the host genome genes.

In the next series of paragraphs, recent studies on the human as well as model organism genomes are discussed as to how they are providing new information about the followings:

- TE diversity,
- biochemical activity arising from TEs,
- how the genome maintains its integrity,
- the balance between TE repression and immune context,
- the challenges that are faced in genomics-based TE studies.

This review concludes in the end as to what the reported findings could mean for future research into genetic variation in human health and disease.

## Recent insights into TE diversity

The human genome project has been transformative in the way we approach genotype-to-phenotype investigations and arguably was the impetus in opening the doors to model organism (genome) research [14]. So we have continued to gain insights by learning the order of the four nucleotide bases within genic regions, but the nucleotide order within repetitive and highly polymorphic stretches of the genome has been less amenable to traditional short-read sequencing approaches [15]. Long-read sequencing (also termed as third-generation sequencing) approaches are now breaking new grounds in understanding complex genetic variations and providing a better grasp on TE activity within human and model organism genomes [16]. A selection of three recent reports discussed below reveals the extent of missing information in terms of TE content and diversity in the ‘reference’ genomes created by short-read-based sequencing approaches [17–19]. These reports are also a testament to the invaluable contribution from model organisms (genomes) in furthering our insight into genetic diversity.

### Population level TE dynamics in *Drosophila*

Rech *et al.* [17] profiled the *Drosophila melanogaster* genome by sampling natural populations from 12 different geographical locations representing five climactic regions: 23 samples from 7 different countries across Europe mainland, 8 from the United States and 1 from South America. In total, 32 genomes were profiled using long-read sequencing (majority Oxford Nanopore Technology platform based) and the resource thus created was used for *de novo* construction and further manual curation to create a library of TE consensus sequences. The present gold standard of *Drosophila* genome annotation, the FlyBase [20], was used to compare and quantify the novelty in terms of genome coverage and TE identification. The authors report identification of 50% more TE copies than in FlyBase. While most additional TE copies belonged to families known in FlyBase, three new TE families were also identified. Thus, a well-studied (and annotated) model organism can also reveal novel TE content if populations from geographically diverse or under different adaptive challenge are profiled via long-read sequencing technologies. When assessed using RNA sequencing, hundreds of *de novo* annotated TEs were found to be associated with either up- or down-regulation of nearby genes. The authors also identified 18 candidate TE insertions with signatures of selection at the DNA sequence level and analysis with previously reported TE insertions revealed developmental and response to stimulus as among the most recurrent biological processes affected by TE insertions.

### Telomere-to-Telomere human genome assembly

In the April 2022 issue of *Science*, the Telomere-to-Telomere (T2T) Consortium presented its results for the end-to-end assembly of the human genome primarily using long-read sequencing of a pseudo-haploid hydatidiform mole cell line, CHM13 [21]. Among the panel of papers published, Hoyt *et al.* [18] present comprehensive repeat element annotation using previously known and *de novo* identified units from T2T-CHM13 genome assembly. In total, 1.65 Gb of sequence content in the T2T-CHM13 assembly (53.9%) was classified as repetitive DNA content and this included a set of 49 previously unidentified repeat types (TEs as well as non-TE). Of the 182.1 Mbp of T2T-CHM13 assembly sequence content with no correspondence within the Genome Reference Consortium human build 38 (GRCh38; current version of reference human genome), 92.4% (168.3 Mbp) are classed as repeat elements. Therefore, the majority of the ‘missing pieces’ in the current genome reference GRCh38 are in terms of repeat elements. Another missing component offered by the new assembly is high-quality annotation of the hitherto unresolved repeat rich centromeric regions for the constituent satellite repeats. The authors also studied nascent transcription and CpG methylation to delineate the transcriptionally active TEs.

### Developmental stage-specific expression of TEs in zebrafish

Zebrafish is a valuable vertebrate model organism like mouse, but with a shorter life cycle and visually accessible early embryonic development. Nonetheless, the zebrafish genome is poorly characterised in terms of TEs, especially so in the light of the fact that though zebrafish and human genomes both are ~50% TE, the retrotransposons account for ~90% of all human TEs but only ~10% in zebrafish [22]. In a recent seminal work, Chang *et al.* [19] present comprehensive annotation of TEs across the zebrafish genome. The authors report that unlike the human genome, many TE families are transpositionally active in the zebrafish genome. Using publicly available bulk and single-cell RNA sequencing data, the authors studied developmental stage-specific TE expression and note that TE-derived reads increased by >4x in post-zygotic genome activation (ZGA) than pre-ZGA. Furthermore, the authors report TE-derived expression clusters specific to different developmental stages post-ZGA. For example, DNA transposons were enriched in late larval expression, in contrast to LTR families that were enriched in earlier developmental stages (post-ZGA). Though single-cell RNA sequence data have lower coverage of the transcriptome repertoire, the authors applied several filters to identify TE-derived signal and identify a small subset of TE families such as the BHIKHARI and ERV1–3 to have high expression in somatic progenitor cell lineages. It can be speculated thus that spatiotemporal patterns of TE expression might have some role in organismal development.

## Evolution of TE origin biochemically active units

The distribution of TEs in the genome is non-random because it is influenced by selection pressure, the specific TE transposition mechanism and host genome distribution of gene dense and heterochromatin regions [4, 23–25]. Among the TEs, Class I and Class II elements have had different success; where Class II elements: DNA transposons comprise only 3% of the human genome, Class I elements: RNA transposons/retrotransposons constitute >30% of the human genome by sequence content. Copy number wise too, the Class I elements are far abundant. Within the Class I TE elements, the long interspersed nucleotide elements (LINE) and short interspersed nucleotide elements (SINE) families are especially noteworthy because of their success in colonising the human genome. Whereas LINEs, possibly due to their large size (in kbs), are enriched in intergenic regions, SINEs have a gene loci-biased distribution [25]. Within the SINE family, Alu elements are particularly poised to contribute to alternative exons in the human transcriptome because of harbouring cryptic splice sites [26, 27]. Such exonisation events (retention of an intron/intron part as an exon or alternative start/end of exon) occur predominantly in the 3′ UTR [27, 28]. In addition to affecting the transcribed exon content, Alu elements also lead to adenosine-to-inosine (A-to-I) editing. This phenomenon is controlled by ADAR class of enzymes acting on double-stranded (ds) RNA substrates in the transcribed content of the cell. Abundant Alu sequences in the intronic region easily create scenario where two Alu elements are in head-to-head orientation (IR-Alu, inverted repeated Alu pairs). Such a situation in pre-mRNA would lead to dsRNA formation and is the most preferred substrate for ADAR enzymes [29–31]. Alteration in A-to-I editing has been reported in multiple pathological conditions and this RNA modification could be used as a modulator of the innate immune response [32, 33].

There is another outcome of IR-Alus, which has relatively recently come into focus and that is the formation of circular RNAs (circRNAs) through back-splicing [34]. As the same sequence construct (IR-Alus) could be the target of RNA editing and facilitator of the back-splicing event, it is interesting to speculate the implications of interaction between the two machineries: ADAR enzymes and splicing regulatory proteins for back-splicing. Indeed, a negative correlation has been reported between ADAR1 expression/activity and circRNA production [35, 36]. Thus, the dsRNA substrate formed by IR-Alus is a hotbed of potential interactions between multiple RNA processing pathways [37].

## Maintenance of genome integrity

TE activity: transcriptional or transposition, around the genome, can be detrimental to the genome architecture of the host or at the least not providing any adaptive advantage to the cellular phenotype. Hence, host genomes have evolved multitude of regulatory mechanisms to keep a check on TE transposition ability and its transcriptional potency. It also serves the subversive occupants well that their host is not affected detrimentally by their presence and biochemical activity.

Epigenetic changes, whether through DNA base modification or through histone proteins, are the most widely used route for TE control [7, 38]. There are multiple pathways and enzymatic machinery that converge to achieve this end point. The P-element-induced wimpy testis-interacting RNAs (piRNAs) is a class of endogenous non-coding small RNA able to distinguish non-self (such as TEs and viruses) and promotes the recruitment of DNA methylation (DNMT) enzymes and/or repressive chromatin marks on TE loci, primarily in germ cells [39–42]. How the piRNAs in complex with newly discovered partner proteins recruit DNA/histone methylating enzymes at the TE loci is still being understood [43, 44]. This is all relevant because during gametogenesis and mammalian pre-implantation development, extensive re-programming of DNMT and chromatin marks is carried out and TEs for a time-period are relieved of genome-wide repression [3]. Among histone methylation marks, the histone modification histone 3 lysine 9 trimethylation (H3K9me3) mark is understood to be the main repressive mark for silencing retrotransposons [45]. More importantly, in mammals, DNA and H3K9 methylation are strongly associated [46]. The link between the DNMT enzymes and the H3K9 mark depositing histone methyltransferase (SETDB1) is Krüppel-associated box (KRAB) domain-containing zinc-finger proteins (KRAB-ZFPs). Humans and mice encode >400 KRAB-ZFPs and majority have sequence-specific recognition for TEs [47]. Once KRAB-ZFPs bind to TEs, co-factors can be recruited and one such co-factor, KRAB-associated protein 1 (KAP1) (encoded by the Trim28 gene), is not only a transcriptional repressor but also crucial for early embryonic development and stem cells [48]. KAP1 can then induce heterochromatin formation (TE repression) by recruiting SETDB1 (histone methylation H3K9 mark) and the resultant protein complex can further promote DNMT by associating DNA methyltransferases. Thus, the genome has evolved an intricate modular toolkit for repressing the TE landscape. The modularity (multiple enzymatic pathways) would allow for fine tuning of the regulatory outcome depending on which of the KRAB-ZFP/methyltransferase members are involved [49, 50]. The methylation marks on TEs during early developmental stages are not necessarily meant for TE repression only. In two recent reports investigating early stages of human embryonic development, H3K9 methylation marks on the hominoid-specific SVA (SINE-VNTR-Alu; composite retrotransposon) elements have been shown to be used as cues for ZGA and lineage segregation (between inner cell mass versus trophoectoderm of blastocyst stage) [51–54]. Indeed, it is reported that CRISPR-KRAB-mediated silencing of certain SVA elements results in defective ZGA and lower rates of blastocyst formation [53].

Given the elaborate regulatory systems in place to keep a check on TE transcriptional activity, it is understandable that an immune response is elicited in the cell when those regulatory checks have not worked. The cellular sensing machinery that works to detect external (like viral) RNA/DNA works for TE activity-related nucleotide fragments too [55]. In case of ds or single-stranded (ss) RNA, retinoic acid-inducible gene I (RIG-I)-like receptors (RLRs), such as RIG-1 and MDA5 that have cytosolic location, perform the detection and subsequent induction of type I interferons (IFNs) and proinflammatory cytokines like IL-6 [56]. Toll-like family receptors can also sense ds/ssRNA and result in similar downstream cascade of type I IFN and inflammatory cytokines [57]. In case of cytoplasmic ds/ss DNA, sensing is performed by the cyclic GMP-AMP synthase (cGAS)-stimulator of interferon genes (STING) pathway to achieve activation of type I IFNs [58], analogous to RLR signalling.

This activation of the innate immune response/type I IFN signalling on sensing aberrant TE activity is observed beyond mammalian genomes too. In zebrafish (*Danio rerio*), larvae harbouring deletion in DNMT machinery, uhrf1 and dnmt1, activated IFN response was observed, and gene expression profile mimicked viral infection [59].

## Balance between TE repression and immune context

Given that epigenetic machinery aims to suppress TEs, failing which (innate) immune system gets activated, this dynamic should have important repercussions in human health conditions where immune system is compromised and/or epigenetic machinery is altered. A prime case in example would be human malignancies where epigenetic landscape regulators are frequently altered [60, 61]. In this context of cancer patients, recent years have seen heightened interest and actual use of immunotherapy-based treatment regimes in the clinic [62]. Nevertheless, patient stratification to decide who would respond the most to immunotherapy and biomarkers that can be used for decision-making has no satisfactory or at least universal answers that could be applicable to a wide variety of solid tissue malignancies [63]. One of the avenues being investigated for making tumours more amenable to immunotherapy is ‘viral mimicry’, i.e*.* induction of type I IFN response from TE origin dsRNA nucleic acid fragments, thus mimicking viral infection in the cell [64]. Griffin *et al.* [65], using CRISPR-Cas9 screens, report KRAB-ZFP—KAP1 repressive complex association with SETDB1 as a mediator of immune escape in tumour. Furthermore, they found that loss of SETDB1 derepresses TE and increases expression of TE-encoded retroviral antigens, which, in turn, triggered cytotoxic T-cell response. In this same context of H3K9 repressive marks on TEs, Zhang *et al.* [66] report loss of KDM5B (a recruiter of the H3K9 methyltransferase SETDB1) to induce robust immune response via activation of cytosolic RNA and DNA-sensing pathways. More recently, Wu *et al.* [67], using an unbiased drug screening for epigenetic inhibitors, identified a type I PRMT inhibitor to be effective across a panel of triple-negative breast cancer cell lines by inducing viral mimicry response through cytoplasmic dsRNA accumulation from retained introns harbouring IR-Alus (inverted repeated Alu elements). This finding should be appreciated in the bigger picture of the downstream consequences for SINE TEs being abundant in intronic sequences and in possession of cryptic splice sites. The potency of forming dsRNA by IR-Alus could not only be immunogenic but is also the substrate for ADAR enzymes for A-to-I RNA editing. More importantly, as ADAR enzyme targeting tends to destabilise IR-Alu originating dsRNA, there could be dependency on ADAR1 for tumour cells undergoing epigenetic therapy. Indeed, Mehdipour *et al.* [68] have shown that for epigenetic therapy that induces transcription of inverted SINEs, ADAR1 depletion potentiates the efficacy of the therapy. Hence it can be postulated that in addition to epigenetic regulator like PRMTs, other pathways of altering the splicing regulatory mechanism could also lead to dsRNA in the cytosol. Indeed, spliceosome-targeted therapies lead to widespread mis-splicing, causing accumulation of dsRNA structures from intron-retained RNAs in the cytoplasm [69].

## Challenges in studying

High-throughput genomic studies using sequencing or microarray-based approaches require sequence uniqueness for any given genomic stretch/gene/transcriptional unit to be assessed individually. Hence, TEs are inherently not amenable to these approaches. Probe design for oligonucleotide arrays avoids repetitive regions of the genome, or parts of gene containing such sequence. Even with whole-genome sequencing or total RNA sequencing, though the total DNA or RNA of the cell might have been in the prepared sequencing library, standard short reads with repetitive sequence/TE content are unable to align uniquely to the genome. Long-read sequencing has been a breakthrough in this regard by allowing us T2T assembly [18, 70] and identification of genetic variation in complex genomic regions [71, 72]. Nonetheless, substantial challenges remain in assessment of functional activity for transposon sequences or repetitive regions in general from the genome.

The abundance of TE units in the genome combined with their polymorphic nature create a challenge in interpreting genetic variation (affecting or arising from TE sequences) in the context of function, i.e. evaluation of genotype to phenotype relations. Genetic variation markers: single-nucleotide polymorphisms (SNPs) or multi-base insertion–deletion (InDels) events or large-scale structural variants (SVs) or copy-number variants, are established tools to study the evolution of phenotypic traits and emergence of disease-causing/linked variation [73, 74]. Such genetic variation when looked at genome-wide scale, and not focussed only within protein-coding regions, cannot escape the influence or at least sequence overlap with the TE content of the genome. Protein-coding genes (exon-intron structure included) are unique (in terms of nucleotide sequence content) stretches of the genome. Variation affecting a particular protein-coding gene is comparatively easier to interpret as compared with a TE family, which could have numerous copies littered around the genome. A good case study is the Alu element belonging to SINE family of TEs. In addition to being present in more than a million copies in the human genome, the individual units can be further subdivided into ‘young’ or ‘old’: the former being relatively newer insertions and also less divergent from consensus (Alu family) sequence [75]. Compared with protein-coding genes, sequence divergence in TEs is more due to lack of positive selection [76]. The distinction between ‘young’ and ‘old’ sub-families of individual TEs is relevant because of the difference in their transposition capability. Within Alu elements, for example, ‘young’ members are transpositionally active and overall, within the human genome, younger TEs, i.e. Alu, L1 and SVA, are transpositionally active [77]. As such polymorphism of TEs is a widespread phenomenon: not only in terms of InDels events but also in terms of sequence divergence within individual TE families. A 2015 report on SVs (defined as ≥50 bp variants, as per the report) identified from 26 different population types (a total of >2500 human genomes) reported >16 600 (~24% of total SVs identified) as mobile element insertions (MEIs) [78]. Such contribution to genetic diversity creates challenge in systemic identification of variants that could potentially be linked to disease phenotype. Mining of GWAS studies could be one approach to identify TE variants linked to human phenotypes. An investigation into Alu polymorphism found that though such events are significantly enriched at GWAS loci, there could be Alu insertion events in linkage disequilibrium with trait-associated SNPs (TAS). The authors identified 44 polymorphic Alu elements at 77 GWAS loci to be correlated with TAS [79]. Recent efforts have aimed to catalogue MEIs in specific population cohorts. A Chinese population-specific cohort reported Alu elements for highest mean insertion sites per donor and a concomitant higher contribution to protein-truncating variants and phenotypic traits (in terms of GWAS SNPs) [80, 81]. More importantly, in the context of MEIs in general, majority of them are rare (minor allele frequency < 0.1%) as reported in the above study and previously [82].

To comprehensively quantify and evaluate such rare events in genomic cohorts is both challenging and in need of exhaustive sequencing approaches, such as long-read platforms. This is especially relevant to overcome the problem of multi-mapping when assigning sequencing reads arising from TEs. To elaborate, short sequencing reads originating from a given TE family can map equally well to the numerous family members spread across the genome (multi-mapping). If one were to counteract this by leaving out multi-mapping TE reads and select only that map uniquely to TE positions, then the quantification output (from an RNA sequencing data set) resembles more the TE family-wise mappability, than the actual transcriptional activity arising from TE families [83]. Mappability is the property of any sequence stretch of the genome that indicates the confidence with which sequencing reads arising from there could be mapped back to it. For example, if a sequencing read arising from a locus has 10 matches in the genome, then the mappability (or alignability) of the locus will be 1/10 = 0.1. As such TEs (and low-complexity regions like microsatellite regions) have low mappability [84, 85]. Recent efforts have focussed on using long-read sequencing to overcome the low resolution of TE loci from short-read sequencing data. Such published data from the human, Drosophila and zebrafish reveal increased TE content (than known before), novel TE families and allow for study of population level TE dynamics [17–19, 86].

**Figure 1:**
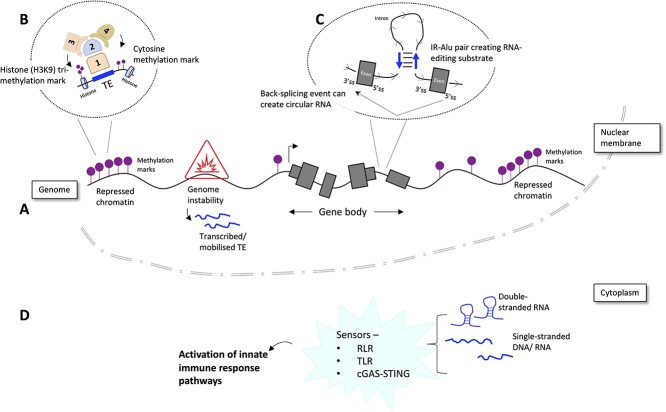
Overview of molecular activity in and around TE loci and its interaction with genome integrity maintenance machinery. (A) Schematic representing genome organisation with heterochromatin regions, a gene body and a genomic instability region. ‘Lollipop’ symbols in purple are methylation marks (DNA cytosine and/or histone methylation). Closely placed methylation markers are indicative of repressed chromatin or heterochromatin. The red triangle with an explosion sign within it is indicative of a genomic region that has become unstable and TEs in that region have become transcriptionally or transpositionally active. The gene body is represented by grey boxes indicative of exons: narrower height towards either end is indicative of the UTRs (untranslated regions). (B) This is a schematic illustrating how repressive methylation marks could be laid on TEs (dark blue rectangle). On top of the TE is an assembly of a protein complex with individual components numbered from 1 to 4: KRAB-ZFP (1), KRAB-ZFP-associated repressor KAP1 (2), histone methyltransferase SETDB1 (3) and DNA methyltransferase DNMT1 (4). The SETDB1 is depositing tri-methylation mark (‘lollipop’ with three overlapping circles) on the histone. And the DNMT is methylating the DNA base cytosine. (C) This schematic shows a would-be scenario in pre-mRNA stage where an inverted repeated Alu (IR-Alu) pair is present in the intervening intron sequence between two exons. The 3′ss and 5′ss mean 3′ and 5′ splice-site, respectively. The sequence complementarity between Alu members is causing the IR-Alu to form a double-stranded RNA (dsRNA) structure. Such dsRNA is the most preferred substrate for the RNA editing enzyme ADAR1. Formation of the dsRNA could have another consequence by helping create the right conditions for circular RNA formation. (D) Schematic representing the cytoplasm where remnants of TE activity such as dsRNA and single-stranded DNA/RNA can be found. These nucleic acids can be sensed variously by RIG-1-like receptor (RLR), toll-like receptor (TLR) and cGAS-STING pathways. Recognition by any of the pathway can lead to downstream activation of type I IFN response (innate immune response).

## Tools and resources

In tandem with increase in read lengths feasible from sequencing platforms, computational tools have evolved to comprehensively quantify the TE landscape, whether from DNA or RNA sequencing. Equally valuable have been TE-specific databases that have catalogued TE family sequences across multiple species/model organisms. The primary step, given a sequencing read in the context of understanding TE biology, is to determine whether the sequence contains a known TE and if yes then what is the family or nomenclature for the detected TE. RepeatMasker [87] has been one of the earliest specialised (consensus sequence based) TE detection tool utilising Dfam [13] and/or Repbase [12] databases of consensus sequences of repeat/TE families. Improving over consensus sequence-based search, RepeatModeler2 has been reported to have *de novo* identification capability for TE families [88]. In fact, in the recently published T2T Consortium data for gapless assemblies of all human chromosomes (except chrY), RepeatModeler2 detected 49 previously unidentified repeat types [18]. *De novo* repeat analysis is becoming increasingly relevant as the cost of sequencing goes down and the length of the sequence read goes up. Detection of MEIs similarly has improved from availability of long-read sequencing platforms, and assessment of TE content within centromeric regions is now viable [18, 89]. Long-read sequencing is being integrated into clinical diagnostics and population specific if not patient specific; genetic landscape is increasingly being referred to in human disorders without clear molecular diagnosis [71, 90]. TEs being a major contributor to genetic diversity, evaluation of available tools is required as sequencing technology improves for TE discovery and annotation. The reader is referred to consult the informative reviews on available repertoire of tools in References [91–94].

Post-human genome, efforts till now have utilised the reference human genome sequence, but as population scale sequencing efforts have proved, a single reference sequence is inadequate in representing the extent of variation seen in different human population types (geographical and/or ethnic). A Human Pangenome Reference Consortium has been created that aims to create ‘pangenomes’, i.e. representative whole-genome sequences per genetically diverse population [95]. As geographical boundaries represent diverse adaptive challenges and genetically divergent population groups have had taken different evolutionary routes, such ‘pangenomes’ would prove invaluable in population-specific assessment of common and rare variants. Another shortcoming in the reference human genome has been the unfilled gaps (bases denoted by letter ‘N’) and unplaced contigs (sequence stretches that have not been able to be fit within the current genome assembly), about 8% of the genome [21]. The T2T Consortium has used long-read sequencing on the genome of a haploid cell line derived from a complete hydatidiform mole (CHM); CHM13. The outcome has been the addition of ~200 Mb of sequence content absent in the current reference genome assembly GRCh38. More importantly, because the T2T-CHM13 genome sequence is gapless and from long-read platforms, a hitherto unreported resolution of TE content in the human genome has been possible [18]. Use of T2T-CHM13 as a reference would allow comprehensive query of genetic variation, especially in complex regions such as TE families and constitutive heterochromatin areas such as the centromeres [96].

Finally, transposon element biology is still a niche field as it has not been long since summary ‘masking’ of repetitive sequences was the norm for sequence-based analysis. With increase in our understanding of genome architecture from human as well as model organisms, our appreciation of repetitive DNA content (whether transposons or low-complexity repeats) is increasing. The TEHub community-oriented resource is an excellent place for newcomers in this field [97]. The specialised research journal ‘Mobile DNA’ covers transposon-related research from any organism and is a valuable resource to remain updated on recent research trends [98].

## Conclusions

The research reports surmised in this review are making connections between regulation of genome integrity, TE activity and how both can interact to change cellular phenotype (Figure 1). Given the understanding we have of genome evolution, the genome (human or any other model organism that we can study) architecture that we observe at present can be thought of as a snapshot instead, of a continuous flow of genetic units colliding, adapting and in the process altering itself and other units. That is to say that the genetic units we are seeing today are an outcome of the past evolutionary adaptation demands. And in the same vein, the genome architecture that shall present in future would have been determined by adaptations that are yet to present themselves. From the different reports on TE evaluation presented in this review, there are some common themes in genome regulation such as conservation against TE insertion within coding sequences. But there also are striking differences in TE landscape across genomes from different species [4]. Like difference in Class I (retro-TE) versus Class II TE (DNA TE) content between the human and zebrafish genome. Or, among teleost fishes, why pufferfish genome has ~5% TE, but zebrafish has >50% content as TE? These processes are operating beyond human observable timescales but research into model organism genomes with smaller lifespan (like in Drosophila) can provide an experimental lab to test out which environmental cues or adaptation challenges could lead to selection for or against TE insertion loci.

This is not an exhaustive review and there are many aspects around TE that could not be included here. One such aspect is the domestication of TE-derived protein sequences via fusion of transposase protein (from DNA transposons) with any host gene; termed host–transposase fusion (HTF). The reader is referred to Cosby *et al.* [99] for a seminal work scanning available tetrapod genomes for putative HTFs. The KRAB-ZFPs, which have been mentioned in this review, are a good example of HTF.

An important consideration when evaluating any genome-wide assay for significant signals is the concept of background activity. Is the transcriptional activity/transcription factor binding-site being observed from/on a TE, more than random chance? And a careful deliberation should be given in deciding what are the thresholds for chance/stochastic event. Without this framework, functional genomic assays could be heavily fraught with false positives [100–102]. Having said that, technological advancements like single-cell and long-read sequencing are allowing us to ask questions that were not feasible in recent past. Evaluation of TE-induced events like ‘viral mimicry’ to modulate patient response would widen the field of research into TEs and bring more ideas on genome evolution in general.

**Key Points**

- Third-generation sequencing approaches and *de novo* TE identification can significantly widen our view of what and how TE copies are evolving.
- Model organism genomes are invaluable tools in assessing TE families under different selection pressure.
- Single-cell RNA sequencing could point out locus-specific TE activity specific to cell type or developmental stage.

## Author Biographies

**Amit K. Mandal:** From studying transposable elements (‘selfish genes’) in PhD, I have moved to study ‘selfish cells’ (tumours). Bioinformatician fellow using data sets from organoids to model cell differentiation routes in colorectal cancer.
